# Interleukin-6 via Sputum Induction as Biomarker of Inflammation for Indoor Particulate Matter among Primary School Children in Klang Valley, Malaysia

**DOI:** 10.5539/gjhs.v5n4p93

**Published:** 2013-04-14

**Authors:** Nazariah S. S. N., Juliana J., Abdah M. A.

**Affiliations:** 1 Department of Community Health, Faculty of Medicine and Health Sciences, Universiti Putra Malaysia, Serdang, Malaysia; 2 Department of Environmental and Occupational Health, Faculty of Medicine and Health Sciences, Universiti Putra Malaysia, Serdang, Selangor, Malaysia; 3 Department of Biomedical Sciences, Faculty of Medicine and Health Sciences, Universiti Putra Malaysia, Serdang, Malaysia

**Keywords:** urban, rural, home, school children, PM_2.5_, PM_10_, IL-6

## Abstract

In the last few years, air within homes have been indicates by various and emerging body as more serious polluted than those outdoor. Prevalence of respiratory inflammation among school children aged 8 and 10 years old attending national primary schools in urban and rural area were conducted in Klang Valley. Two population studies drawn from the questionnaires were used to investigate the association between indoor particulate matter (PM_2.5_ & PM_10_) in a home environment and respiratory implication through the understanding of biological responses. Approximately 430 healthy school children of Standard 2 and Standard 5 were selected. Indication of respiratory symptoms using adaptation questionnaire from American Thoracic Society (1978). Sputum sample collection taken for biological analysis. IL-6 then was analyse by using ELISA techniques. Indoor PM_2.5_ and PM_10_ were measured using Dust Trak Aerosol Monitor. The mean concentration of PM_2.5_ (45.38 µg/m^^3^^) and PM_10_ (80.07 µg/m^^3^^) in urban home environment is significantly higher compared to those in rural residential area (p=0.001). Similar trend also shows by the prevalence of respiratory symptom. Association were found with PM_2.5_ and PM_10_ with the level of IL-6 among school children. A greater exposure to PM_2.5_ and PM_10_ are associated with higher expression of IL-6 level suggesting that the concentration of indoor particulate in urban density area significantly influence the health of children.

## 1. Introduction

The science of indoor air pollution is not relatively new, however it is still developing. In recent years, indoor air quality had caught attention of researchers and publics. Episode of great air pollution in highly industrialized and urbanized areas has made people realized to the quality of air they breathe (Burroughs & Hansen, 2004). It is well known that indoors concentrations of several pollutants can be many times higher compared to those of outdoors. Particulate matter is a collective term used to describe small solid and liquid particles that are present in the atmosphere over relatively brief to extended periods of time (Godish, 1997). These particles originate from a variety of sources. The coarse particles (PM_10_) are mostly produced by mechanical processes, industrial cutting, grinding and also by human activities. In contrast, the fine particles (PM_2.5_) in the urban area of aerosol is dominated by anthropogenic emissions (Mon & Becker, 1999) and come from combustion sources especially from automobile exhaust (Kleinman, 2000). Furthermore, World Health Organization (WHO) in 2002 reported that the effects of indoor air pollution caused approximately 2.7% of the global burden disease worldwide.

With this concern children are among the most susceptible to the adverse effects of air pollution Previous studies have highlighted that children are experiencing the process of growing, along with their body, children's lung may also develop and changing (Yassi et al., 2001; Kleinman, 2000) as well as their immune system which are not fully developed (Hirsch, 2009; Smith et al., 2000). Thus, this make they are more sensitive than adults. This reaction may be elevated as children spend more times indoors leading to a greater risk if compared to adults (Ismail et al., 2010). The environment in which children lives may contribute to a children's risk of having respiratory symptoms or even diseases. Smith (1993) recommending the importance of examining pollutants where people spend most time, as well as in the places where the ambient levels are high. This suggesting to examine the home environments as it is one of the most place where the children spend most of their time. Burroughs and Hansen (2004) also pointed that indoors environment can play a critical role in exposure levels and health outcomes. Thus, it has become increasingly evident that the levels of many pollutants may be higher inside buildings than outside. Epidemiological studies report on home indoor environment on contributing to air contaminants from outdoor and indoors sources from smoking, gas heating and indoor cleaning activities releasing particulate matter and other pollutants thus, making it as a relevant for studying children airway inflammation (Colbeck et al., 2010; Dasgupta et al., 2006; Abt et al., 2000; Thantcher & Layton, 1995). Epidemiological studies in the last few decades have been observed a significant linked between indoor pollutants affecting children's respiratory system (Fujii et al., 2001; Monn & Becker, 1999; Qian et al., 2004; Smith et al., 2000).

Malaysia currently is experiencing rapid urbanization (Salleh, 2006; Zakaria, 2007), however, the growth of the urban population was much more rapid than the population growth in rural areas (Jaafar, 2004). Urban which contribute a high level of air pollution has showed a positive linked with the production of cytokine in alveolar marchophage (Yoshida et al., 2010; Calcabrini et al., 2004). This also showed by a few studies suggested that children in rural areas reported less respiratory symptoms and other sensitization (Braun-Fahrlander et al., 2001; Zhou, 2006) compared to those in urban areas. Indoor environment in Malaysia is one of the particular interest to study for various reasons especially for the prevalence of respiratory symptoms and illnesses as Malaysia is a tropical country with a lower ventilation rates. However, uptill now no regulation to be advice on indoor air qualities (IAQ) in Malaysia as it is currently in developing to have a good environmental law.

In order to understand the relationship of indoor air exposure between children by different area and inflammation on their lung, this study used biomarkers as a surrogate for biological responses towards indoor air pollutants. The biomarkers involved is a pro-inflammatory cytokines which is Interleukin-6 (IL-6). This pro-inflammatory cytokines has been associated to a number of diseases including systemic reactions (Kishimoto et al., 2006) and known as mediator of inflammation especially in pathogenesis of lung diseases (Ricon & Irvin, 2012; Pedroza et al., 2011; Tasaka et al., 2010). A great body of scientific evidence reporting on indoor air pollutants with the changes in respiratory health of children, but generally focused on asthmatic children, animal or using cell-medium studies. Thus, not much study was found to actually relate the effects of indoor air pollutants with children without respiratory illness by using biomarkers as indicator. Limited studies on cytokine production of lung inflammation were compared especially in relating with IL-6 among healthy children in different areas.

## 2. Methodology

As a developing area, Klang Valley has become a major source of air pollution. Two areas were select in Klang Valley represent urban and rural community to observe the different pollution level. According to Department of Statistic (2010), urban is referred as the special development area that can be identified, which at least had a population of 10,000 with at least 60% of the population (aged 15 years and above) were involved in non-agricultural activities. While rural in this present study used the definition by Herzog and Pittman (1995) which straightforward where an area which is not a city or urban or non-metropolitan area. From there, a list of grade A classified National Primary School were acquired from Ministry of Health with more than 1000 children per school. Two schools represent urban while one school in rural area were selected randomly.

### 2.1 Selection of Respondents

A total of 430 children were recruited from 4 schools in Standard 2 (8 years old) and Standard 5 (10 years old) who had never been diagnosed with any respiratory. The selection was based from the name list obtained from school medical records. However, the selection was not representing a general school distribution in Malaysia due to larger number or Malays students in national primary school thus, restrict the study to select any representatives of Chinese and Indian backgrounds. Estimation of sample size (23%) was refered to the prevalence of cough among the school children in the study by Juliana (2004). Then, each children selected were purposively sampled based on the distance of their house less than 5 km radius from their school to minimize the potential confounder that might subjectively to the results validity of the study. The selection being made to remove the possibility of other factors affected the subjects and also to homogenize the air pollutants ambient.

### 2.2 Ethical Approval

Approval was obtained from the Committee of Ethics of Faculty of Medicine and Health Sciences, University Putra Malaysia. To carry out this study, a permission was acquired from Malaysian Ministry of Education. As well as approvals from the respective schools obtained for their concern as to ensure the data collection was done during appropriate time. A written consent from parents or guardians was obtained prior data collection with all of information gathered were protected in all phase of study. Sputum sample collected from the children were gained only after getting the parents’ permission.

### 2.3 Questionnaires

In the early phase, the possible subjects for this study were given standardized questionnaire adapted from American Thoracic Society (1978). Questionnaire consists of variables, which has been reviewed in the literature, such as; socio-demographic information, house condition and location, lung disease history, respiratory symptoms (cough, chronic cough, phlegm, chronic phlegm and chest tightness, wheezing) and family background. The children returned the questionnaires that were completed by parents or guardian within 2 days. Any returned questionnaire which does not complete answers were contacted by the researcher and interview them during the measurement process in their home.

### 2.4 Sampling

Indoor air measurement was done in the children’ homes specifically in the living room. The living room is known where the family spent most of their time. TSI DustTrak Aerosol Monitor is a direct reading instrument air monitoring tool used to measure aerosol with an upper particle size limit of 10 µm. Through the DustTrak monitor, the flowrate always be set at 1.7 liters per minute (L/min) with range of detection is 0.001 to 100 mg/m^3^. Thus, this DustTrak was placed at a height of 1 meter above the ground and 1.5 meters (Colbeck et al., 2010) away from doors and windows. Prior to monitoring, a briefing on the procedures and the importance to follow the procedures were given to the parents and respondents in order to avoid any misused of the instruments. The measurement was done for an average of 8 hours due to the limitation which referred to the quantity of DustTrak used in this study. The availability of this equipment was one unit per house which made it as the study limitation.

### 2.5 Sputum Samples

IL-6 were identified using sputum taken from school children which collected using an induction procedure via an ultrasonic nebulizer (Ultrasonic Nebulizer, Model CUN60, Citizen Systems Japan Co Ltd) where coughing is induced by inhaling salt water vapor or hypertonic saline. Sputum samples were collected during school hours upon granted permission by parent or guardian and return of completed questionnaire. Sputum induction has been proven as a successful techniques applied from normal or healthy children (Gibson, 1998). Samples were collected in a specimen bottle before being transported and stored at - 80°C in the laboratory.

### 2.6 Human Interkeukin-6 (IL-6)

Enzyme-Linked Immunosorbent Assay (ELISA or EIA) were used for analyzing IL-6 concentration levels from sputum samples taken from the school children. IL-6 concentration was quantified after incubation with detection of antibody. Collected sputum was ultracentrifuge for 8,000 × *g* for 20 min 4°C (Delacourt et al., 2002) and kept under -80°C until the sample were analyzed. Analysis sputum of human IL-6 were carried out in Chemical Pathological Laboratory, Department of Pathology, University Putra Malaysia.

### 2.7 Statistical Analysis

Indoor air pollutants concentration were analyzed with and the level interleukin-6 (IL-6) according to the area of residence. The comparisons between two areas were tested using independent t-test. To explore the main factor influencing the biomarkers inflammation exposed to indoor particulate matter, multivariate analysis were used in which the potential confounding factors were controlled. All statistical analyses were performed using the Statistical Packages for Social Sciences (SPSS).

## 3. Results

### 3.1 Socio-Demographic Information

The distributions of background information are shown in Tables 1. Table 1 provides the comparison of socio-demographics background and socio-economic status of the samples between two areas. Socio-demographic in the table showed significantly differences for parental education level, household income and the type of housing area between schoolchildren who live in urban area and those living in rural area.

**Table 1:** Distribution of backgrounds among selected school children Klang Valley

Variables	Urban (n=232) Number (%)	Rural (n=198) Number (%)	χ^2^/z value	P value
**Education Level (Father)**			126.19^a^	0.000**
Primary education	1 (4)	25 (12.6)		
Secondary education	108 (46.6)	141 (71.2)		
Higher education	123 (53)	32 (16.2)		
**Education Level (Mother)**			98.85^a^	0.000**
Primary education	3 (1.3)	40 (20.2)		
Secondary education	141 (60.8)	130 (65.7)		
Higher education	88 (37.9)	28 (14.1)		
**Types of housing area**			57.63^a^	0.000**
Villa	14 (6)	3 (1.5)		
Apartment	92 (39.7)	9 (4.5)		
Single storey terrace	50 (21.6)	97 (49.0)		
Double storey terrace	76 (32.8)	24 (12.1)		
Village	0 (0)	65 (32.8)		
**Socio-economic**				
Total income (RM)	4700 (3500)	2275 (1500)	- 11.69^b^	0.000**
Total dweller	5 (1)	6 (1)	- 2.15^b^	0.031*
Total room	3 (1)	3 (1)	-4.19^b^	0.001*
Crowding ratio	1.7 (0.7)	1.8 (0.5)	-0.039^b^	0.969

** Significant at p<0.001

* Significant at p<0.05

a Chi-square test

b Man U Whitney Test

N=430

Majority of the school children's parents in urban and rural areas were a secondary education level which they ended their study on Form 5 or diploma level as refer to Malaysia education system. The statistical results obtained demonstrated that fathers’ and mothers’ highest education level showed significance difference between urban and rural area, respectively.

This study explore the comparison of socioeconomic background and found mean of total income for urban and rural area was significantly difference with RM (5463.32 ± 3455.55) and RM (2376.46 ± 1189.28), respectively. The table also shows the comparison of the total dweller, which refer to the number of person living in a house, and also total room which refer to the number of rooms available in the house. From that, total rooms is divided with total dweller in a house and calculate to get crowing ratio (Hall, 2010). There were significant differences in all variables for socioeconomic background (p<0.05) except for the crowding ratio.

### 3.2 Indoor Air Levels of PM_2.5_, & PM_10_ in Home of Urban and Rural

Table 2 shows a comparison of concentration of indoor air pollutants levels of PM_2.5_ and PM_10_ between homes in urban and rural areas. The data available by measuring the concentration of PM_2.5_ and PM_10_ using air sampling monitoring (DustTrak). From the statistical analysis, mean value of PM_2.5_ concentration in urban residential area was (50.77 ± 10.93 µg/m^3^) significantly nearly two times higher than the mean value of indoor PM_2.5_ concentration in rural area (25.63 ± 8.66 µg/m^3^). Comparison of indoor aerosol levels for PM_10_ in home environment showed similar tabulation between the two areas. Mean indoor PM_10_ in urban (80.07 ± 11.03 μg/m^3^) homes was significantly greater than those living in rural (45.38 ± 10.48 μg/m^3^) area (p ≤ 0.001).

**Table 2:** Comparison of indoor air pollutants inside home environments

Variables	Urban (n=232) Number (%)	Rural (n=198) Number (%)	z Value	p Value

	Median (IQR)	Median (IQR)		
PM_2.5_ (μg/m^3^)	48.96 (12.55)	25.71 (15.72)	-17.12	0.001**
PM_10_ (μg/m^3^)	80.3 (12.99)	43.66 (15.5)	-17.29	0.001**
IL-6 (pg/ml)	3.89 (2.35)	3.53 (2.42)	-2.29	0.02*

** Significant at p<0.001; IQR = Interquatile range

### 3.3 Comparison of IL-6 Level

Biomarkers were used as surrogate to understand the physiology of pulmonary inflammation affected by indoor air pollutants. The marker used is IL-6 as it is one of the cytokine known for it role in the respiratory inflammation. The sputum samples taken from the children were being analyzed for IL-6 concentration levels. According to the statistical analysis, IL-6 level shows a significant difference between children in urban and rural areas (Table 2). Mean of IL-6 concentration among children in urban area determined as 4.27 ± 2.03 pg/ml which slightly higher compared to those in rural areas (3.77 ± 1.67 pg/ml). Comparison between these two study groups were observed with significant difference (p = 0.02).

### 3.4 Prevalence of Respiratory Symptoms

Respiratory symptoms were identified using the questionnaire which was answered by parent or guardian. The parameters for respiratory symptoms studied were cough, phlegm, wheezing and chest tigthness. Indoor PM_2.5_ concentration inside the houses were categorized into high and low value by using above (≥ 38.934 μg/m^3^) and below (< 38.94 μg/m^3^). Meanwhile, indoor PM_10_ concentration was also categorized by using median value with high level and low indoor level (≥ 65.93 μg/m^3^ and < 65.93 μg/m^3^, respectively). A correlation test was conducted and the statistical analysis showed a significant relationship between indoor PM_2.5_ concentration in homes and all respiratory symptoms (cough OR= 1.64, CI 95% = 1.07-2.53; phlegm OR= 1.82, CI 95% = 1.82 -3.08 & wheezing OR= 4.45, CI 95% = 1.89 -10.43) except for chest tightness. A similar trend with previous indoor PM_2.5_ were establish for indoor PM_10_ concentration (Table 3) with significant relationship showed for cough (OR= 1.81, CI 95% = 1.18-2.79), phlegm (OR= 2.45, CI 95% = 1.42-4.24) & wheezing (OR= 5.43, CI 95% = 2.21-13.37).

**Table 3:** Correlation between indoor PM_2.5_ in homes and the prevalence of respiratory symptoms

Variables	Frequency (%)		χ^2^ value	p-value	OR	95% CI

	High indoor level (n=215)	Low indoor level (n=215)				
**Cough**			5.18	0.03	1.644	1.07-2.53
Yes	69 (32.1)^a^	48 (22.3)^b^				
No	146 (67.9)	167 (77.7)				
**Phlegm**			4.99	0.035	1.817	1.82-3.08
Yes	43 (20)^a^	26 (12.1)^b^				
No	172 (80)	189 (87.9)				
**Wheezing**			13.72	0.001	4.449	1.89-10.43
Yes	28 (13)^a^	7 (3.3)^b^				
No	187 (87)	208 (96.7)				
**Chest Tightness**			2.01	0.499	-	-
Yes	0 (0)^a^	2 (0.9)^b^				
No	215 (100)	213 (99.1)				
**Cough**			6.21	0.017*	1.73	1.12-2.66
Yes	70 (32.6)^c^	47 (21.9)^d^				
No	145 (67.4)	168 (78.1)				
**Phlegm**			10.79	0.001**	2.45	1.42-4.24
Yes	47 (21.9)^c^	22 (10.2)^d^				
No	168 (78.1)	193 (89.8)				
**Wheezing**			13.72	0.001**	4.45	1.89-10.43
Yes	28 (13)^c^	7 (3.3)^d^				
No	187 (87)	208 (96.7)				
**Chest Tightness**			2.01	0.499	-	-
Yes	0 (0)^c^	2 (0.9)^d^				
No	215 (100)	213 (99.1)				

N=430

a High indoor PM_2-5_ level

b Low indoor PM_2-5_ level

c High indoor PM_10_ level

d Low indoor PM_10_ level Odds ratio significant at Confident Interval 95% >1

### 3.5 Association between Indoor Air Concentration Parameters and IL-6 Level

Statistical analysis verified that the association between indoor air particulate with the production of IL-6 level among school children were significantly associate. The result observed both particulate matter (PM_2.5_ and PM_10_) shows strong linked with the level of IL-6 in respect to the pollution level.

**Table 4:** Correlation between concentration of Indoor air pollutants and IL-6 level

Variable	Urban (n=232)		Rural (n=198)	

	r value	p value	r value	p value
PM_10_ (μg/m^3^)	0.599	0.001**	0.419	0.001**
PM_2.5_ (μg/m^3^)	0.506	0.001**	0.519	0.001**

** Significant at p<0.001

### 3.6 Factors Influencing the Concentration of IL-6 among School Children

Multivariate analysis using linear regressions performed to determine the main factor contributing towards IL-6 expression in school children. The analysis included exposure level of PM_2.5_ and PM_10_ of respondents. Results from analysis revealed that the exposure level of PM_2.5_ and PM_10,_ recorded significantly influenced the concentration level of IL-6 among the school children.

**Table 5:** Factors influencing concentration IL-6 among school children

	Coefficient Regression (β)	t	p
**Constant**	1.509	5.327	0.000**
**PM_10_**	0.027	3.944	0.000**
**PM_2.5_**	0.022	2.564	0.011*
**Smoker in house**	0.093	2.407	0.017*
**Total Income**	0.000	-3.165	0.002*

* Significant at p < 0.05;

** Significant at p < 0.001

R^2^ = 0.194 (adjusted R^2^ = 0.188) N = 396

## 4. Discussion

A significant difference found in the indoor levels of PM_2.5_ and PM_10_ with range in every homes measured in urban area was higher than the indoor levels found in rural area. The data taken in urban was exceeded the permissible limit by two international threshold standard establish by EPA for primary 24-hour standard level of 35 μg/m^3^ (USEPA, 2012) while, WHO Air Quality Guidelines of 24-hour period exposure (25 μg/m^3^) (WHO (2005). Meanwhile, indoor PM_10_ level in urban residential is within the permissible value (150 µg/m^3^) suggested by the 24-hour exposure Recommended Malaysia Ambient Air Quality Guidelines (DOE, 1988), 8-hour exposure Malaysia Code of practice (COP) (DOSH, 2010) and EPA 24-hour PM_10_ guideline (USEPA, 2012b). The significant difference between indoor PM_2.5_ and PM_10_ concentrations suggests that the local surroundings might influence the concentration of the particulate in the residential of urban areas. Malaysia is a developing country which undergoing urbanization and industrialization, thus the indoor air quality is strongly influenced by motor vehicles emission, however, industrial sources have been control by selecting urban area with no industrial activities surrounding.

Due to the close proximity of the main road from residential area, traffic emission pollutants were easily entered the homes via doors and windows as majority of houses in urban used open ventilation system.

The factors help to contribute and generate higher indoor pollutants and allowing greater risk to the children. According to statistic data in Malaysia by DOE (1996), at least 70% to 75% of the total air pollution were majorly contribute by the emissions from traffic vehicles.

Indoors pollution sources in homes release particles into the air are one of the main causes of indoor air quality problems. There are many factors that may affect the concentration of these particles in the homes especially the activities inside the house. Activities involving cooking and frying may contribute to the increase of high particulate matter in the house (Dasgupta et al., 2006; Wallace et al., 2003). Abt et al. (2000) claimed that cooking inside home contributed 25% of both indoor PM_2.5_ and indoor PM_10_ by a research group of The Particle Total Exposure Assessment Methodology (PTEAM) (Ozkaynak et al., 1996 cited in Abt et al., 2000). This present study analyze the frequency of cooking which may influence the accumulation of particles inside homes thus, from the meeting with the parents, 54.3% of them living in an urban residential area performed cooking twice per day while, 60% of the parents in rural areas cook only once a day (data not showed). The higher frequency of cooking activities in urban is because mother is a housewife and some of the family hire assistance to handle the household matter. Therefore, it is possible for the fine particles especially, to accumulate indoors and suggesting a greater levels of fine particle indoors.

Cleaning activities and indoor occupancy may also play a key role in the increased of particulate levels in the living room (Thatcher & Layton, 1995; Colbeck et al., 2010). Simple activities as walking in and out of the rooms may elevate the mass of particles especially course fraction which suspended by almost 100% (Thatcher & Layton, 1995). This movement involving any human activities contributes to the resuspension of the indoors particles level as demonstrated by Clayton et al. (1993), which also agreed by (Thatcher & Layton, 1995) demonstated that the resuspension rate is highly dependent on activity and particles fraction and thus, can be expected to vary widely. Conjunction with this action, larger particles commonly involved and at it high peak suggesting it as one of the dominant source indoors (Jones et al., 2000). A study by Colbeck et al. (2010) discussed on similar issues, however, indicates opposite outcome by revealing that indoor levels of particulate matter were higher in rural areas. Measured a 24 hours average indoor concentration of PM_2.5_, PM_10_ and PM_1_ in the living room reported indoor concentration range within 550 to 950 µg/m^3^ and 350 to 500 µg/m^3^ for rural and urban residential areas, respectively. Cleaning activities through this present study also in line with previous discussed findings stating fine particle contribute from the housework.

Colbeck et al. (2010) also highlighted besides cooking, those indoor activities such as smoking and watching television might also resulted in the suspension of particulate matter in the living rooms. These factors were observed and found to be one of the determinants for dispersion of particulates matter in the house. Osman et al. (2007) reported average indoor PM_2.5_ levels were 18 mg/m^3^, which was significantly higher in smoking households (p ≤ 0.001). This appeared to be smoking by visitors which also showed a significant cause of PM_2.5_ in non-smoking households. Similar report showed by Wallace et al. (2003), confirm that smoking as the principal contributor to indoor pollution with mean indoor value in the 101 smoking homes was 46.5 μg/m^3^, compared with 17.8 μg/m^3^ in the 193 nonsmoking homes.

This present study also assessed the prevalence of respiratory symptoms and indoor air pollutants inside homes. The statistical analysis showed both course and fine particulate matter (PM_2.5_ & PM_10_) were significantly correlate with all respiratory symptoms except for chest tightness. Due to higher level of PM_2.5_ and PM_10_ in urban might increase a chance of health problems among the school children. Extensive epidemiological studies evidence supports the association of particulate matter with the prevalence of respiratory symptoms which might lead to adverse health effects and mortality (Ostro et al., 2007; Nicolai et al., 2003). McCormack (2009) conducted a study in Baltimore has demonstrated that among children with asthma or other respiratory symptoms indicates that those living in the urban residence had indoor particulate matter concentrations that were two to three times greater than those in the local suburban. While, Clarke et al., (1999) observed increments of approximately 15 μg/m^3^ in urban areas of urban area in comparison with a rural area, at where PM_10_ concentrations were on average 70% of those at urban area. A cross sectional study by Braun-Fahrlander and colleagues (Braun-Fahrlander et al., 1997) conducted among school children aged between 6 to 15 years in ten different areas of Switzerland determine a significant risk of bronchitis found in the most polluted area (OR=2.17) with PM_10_.

This present study also found similar result on both particles fine and course fraction to have a close linked with the prevalence of respiratory symptoms. Extensive epidemiological studies evidence supports the association of particulate matter with the prevalence of respiratory symptoms which might lead to adverse health effects and mortality (Ostro et al., 2007; Nicolai et al., 2003). McCormack (2009) conducted a study in Baltimore has demonstrated that among children with asthma or other respiratory symptoms indicates that those living in the urban residence had indoor particulate matter concentrations that were two to three times greater than those in the local suburban. While, Clarke et al. (1999) observed increments of approximately 15 μg/m^3^ in urban areas of urban area in comparison with a rural area, at where PM_10_ concentrations were on average 70% of those at urban area.

Accordance to a few studies found an association between those resides within close proximity to traffic density may potentially increase the airways inflammation among children (Nicolai et al., 2003; Janssen et al., 2001; Ciccone et al., 1998; Chen et al., 1998; Brunekreef et al., 1997; Oosterlee et al., 1996). To assess the impact this traffic emission, PM_2.5_ and NO_2_ are mostly considered as the two air pollutants marker for traffic pollution (Cyrys et al., 2005; Heinrich & Wichmann, 2004; Studnicka et al., 1997).

A cross-sectional study by Van Vliet et al. (1997) conducted among 1068 children in Netherlands found that respiratory symptoms of cough, runny nose, wheeze and doctor-diagnosed respiratory illness were significantly more often reported for children living within 100 meters of a main road. While, Venn and collegeous in 2001 lead a case-control study in Nottingham, England and discovered that among 6147 children were potentially to risk of wheeze linked especially with those living within 90 meters from the main road (Venn et al., 2001).

A panel study (CPCD, 2008) conducted in India for 3 months had comparing the exposure to air pollutants in Delhi (urban) and rural areas of Uttaranchal and West Bengal among school children aged between 9 to 17 years. Children in Delhi had 2 times more (17% vs. 8%) lower respiratory symptoms (LRS) includes frequent dry cough, sputum-producing cough, wheezing, breathlessness, chest tightness and sleep disturbance due to breathing problems. Thus, compared to rural areas, children in Delhi had 1.8 times more upper respiratory symptoms (URS) and 2 times more LRS suggesting a higher prevalence of underlying respiratory symptoms. The prevalence of respiratory symptoms among children of Delhi also correlated with the level of PM_10_.

Epidemiologically, IL-6 is one of the first cytokines to be associated to particulate matter exposure (Alfaro-Moreno et al., 2002). This present study collects a total of 396 sputum samples induced from school children indicating those living in urban area was significantly higher (z= -2.29, p= 0.022) than the children living in rural area. This may concludes that the school children exposed to higher indoor air pollutants significantly express a higher level of IL-6 with strong associaton found between the variables (p= 0.001).

A few epidemiological studies addressed a comparable finding with this present study suggesting an increase of PM_2.5_ (Calcabrini et al., 2004; Schwartz et al., 1996) and PM_10_ (Hetland et al., 2004; Becker et al., 2003), may have the potential to induce the release of pro-inflammatory cytokines. Similar trend found in a study by Alfaro-Moreno (2002) suggesting that particles from the central zone of Mexico City were the most potent in causing cytokine secretion. Measured by using TNF-α and IL-6, both cytokine production had a similar trend indicating that particles from the central (urban) zone were more powerful than were particles from northern (industrial) and southern (residential) zones. Though, no statistical significant showed for TNF-α however, in IL-6 case differences were statistically significant (*p* < 0.0001) found. Maximal TNF-α and IL-6 average secretion observed with particles from the center was 37.7 ± 8.4% and 7.1 ± 0.3%, respectively.

Eventhough, both fine and course fraction particles were seen to associate with the high level of IL-6 production, the multivariate analysis in this present study however, demonstrated that PM_10_ mostly linked with the expression of IL-6 in the school children. However, a scrawny value showed in the table testifying a weak predictor by these solely indoor particulate matters. This may contribute by the presence of other factors such as total income which may reflex the capability of the family to acquire health services for their children. Beside, presence of smoker in the house has been showed to have an association with the concentration of fine particles in the house (data not showed), suggesting that PM_2.5_ release from smoking activitry also become one of the factor which might influence the higher concentration level of IL-6 in children.

A strong association has been found more on the course fraction. Epidemiologic evidence including *in vivo* and *in vitro* reported in several regions in research of fine and course particles signifying that the course particle (PM_10_) were greater risk for cytokine release and better inducers of cell death from the same area (Pozzi et al., 2003; Becker et al., 2003; Monn & Becker, 1999). The effect also investigated by Hetland et al. (2005) which the ability of the particle samples to induce release of the pro-inflammatory cytokines TNF-α and IL-6 from rats. According to the study, coarse fractions were more potent to induce the inflammatory cytokines TNF-α and IL-6 than the corresponding fine fractions. However, Xu et al. (2008) explored TNF-α and IL-6 with PM_2.5_ and indicate that this fine particle has the ability to impair the phagocytosis function of AM, which this might be the mechanism of chronic pulmonary diseases. Besides, Calcabrini and colleagues previously demonstrate TNF-α and IL-6 release in dose- and time-dependent manner after exposure to PM_2.5_ (Calcabrini et al., 2004). Therefore, size of the particles generated in homes which reflected by their formation processes may induce different response by the lung cell (Orsornio-Vargas et al., 2003; Ovrevik et al., 2005).

This study suggested that school children who live in urban area of Klang Valley were exposed to a higher level of indoor particulate pollutants inside home environment. Both particulate matter reported as one of the contributor factor to the increase level of IL-6 measured through sputum samples. Therefore, IL-6 can be suggested as a biomarkers for the respiratory problems in a healthy children with the used of sputum induction. However, this particulate matter cannot be solely predictor to the production of IL-6 in respect to respiratory implication. But, if the children continuously exposed for a longer period of time, toghether with other indoor sources may potentially interfere with the development of children's lung. Besides that, more research into pulmonary cytokine production should be carried out for further understanding of lung inflammation especially among children with sputum techniques. Further research should also be conducted in the future including measurements of other indoor pollutants and particles as well as their mixture component in order to determine in detail the significance differences with a large number of sample sizes. In addition, indoor sources should also be considered and need to be investigate further in order to understand their effects to children health. Limited studies found for sputum techniques used among children for biomarkers analysis which suggests that a broader understanding needed to understand the mechanism relating to the biomarker of inflammation.

## 5. Study Limitation

There may be some information bias happened which is recall bias as the parents or guardians may not recall well the actual incidents happen to their children. Furthermore, information or measurement bias may occur in filling up the questionnaire where some of the parents or guardian may falsely give information due to diverse understanding. Besides that, to assume the parents or guardian answer the questionnaire truly and honest may lead to a limitation as the parents or guardian may in any way lie or being not sincere in answering the questionnaire as result to protect their concerns.
